# A Prospective Study of Burn Trauma in Adults at the University of Calabar Teaching Hospital, Calabar (South Eastern Nigeria)

**Published:** 2008-07-21

**Authors:** Maurice E. Asuquo, R. Ekpo, Ogbu Ngim, C. Agbor

**Affiliations:** Department of Surgery, University of Calabar Teaching Hospital, Calabar, Nigeria

## Abstract

**Background:** Burn injuries are among the most devastating injuries seen in the emergency units. The epidemiology of this injury varies from one part of the world to another. This is a 3-year report in an attempt to provide information on the current epidemiology of burns in this center. **Method:** Patients admitted into the University of Calabar Teaching Hospital, with burn injuries were prospectively studied between February 2005 and January 2008. **Results:** The 59 patients (33 males and 26 females) accounted for 3.7% of trauma patients and their ages ranged from 15 to 70 years (mean 29.4 years). Flame burn was the commonest injury seen in 48 (81.3%) patients because of petrol and kerosene, whereas chemical burn that involved 7 (11.9%) patients ranked second. Morbidity included burns wound infection in 13 (22%) patients and contractures in 6 (10.2%) patients. The outcome was fatal in 15 (25.4%) patients. **Conclusion:** The establishment of burn support groups dedicated to publicity on prevention based on areas of risk highlighted in this study and provision of financial aid as well as provision of modern burn care facilities would improve outcome.

Burn injuries are among the most devastating injuries seen in the emergency unit ranging from minor to lethal injury. Excluding road traffic injuries, they are the most common cause of accidental deaths in both the developed and developing countries. The past few decades have seen many changes in burn care aimed at decreasing patient morbidity and mortality.^1^ The establishment of improved resuscitation, specialized burns care centers, early surgery, nutritional support, and skin replacement techniques have decreased morbidity and mortality.^2^ The cost of managing these injuries is high, and most developing countries including Nigeria cannot afford the high cost of providing modern burns care facilities.^3^

Burns often result in severe deformity, disability, and adverse psychological reactions, which affect patients and their parents.^4^ The cost of managing these injuries is high, worse still are the poor facilities in most parts of the developing countries including Nigeria.^3^

The epidemiology of this injury varies from one part of the world to another and even in the same environment over a time period. It is a function of civilization, industrialization, culture, and societal stability.^3^,^5^ From February 2005, we undertook a prospective study of burns trauma as part of a wider prospective study of the University of Calabar Teaching Hospital, Trauma Research group, headed by professor Bassey. This communication is a 3-year report (February 2005–January 2008) of this ongoing study, in an attempt to provide information of the current epidemiology of burns in this center, its morbidity, mortality profile, and recommend appropriate measures for improved outcomes of management and prevention.

## PATIENTS AND METHODS

All the patients managed in the University of Calabar Teaching Hospital, Calabar, with burns injury from February 2005 to January 2008, were prospectively studied based on a questionnaire. This included, biographic data, social history, associated medical conditions, mechanism/circumstance of injury, prehospital care, primary/secondary survey, associated injury, investigations, and initial and definitive treatment. The incidence was compared with total number of emergencies and traumatic injuries recorded during the same period.

On admission, the ABC of resuscitation was instituted. The assessment of extent of burns were based on Wallace's rule of 9. The size of the palm, estimated at 1%, was used to calculate the extent of burns especially when sparsely distributed. The depth of burn was categorized into partial and full thickness. *Partial-thickness* burns were further subcategorized based on the clinical findings, into first degree (epidermal burn)—as painful erythema; second degree (superficial dermal burn)—painful blisters and bright red raw surface; and deep dermal—blister may have ruptured with cream colored or mottled appearance. The *third-degree* or *full-thickness* burn was defined as absence of pain and blisters and gray or white charred appearance (eschar).

Except for mild inhalational injury, patients with stridor, hoarseness, facial/circumoral burn, tachypnoea, and confused or aggressive behavior (may be due to cerebral hypoxia) were admitted to intensive care unit. Others included severe burns with haemodynamic instability, refractory to resuscitation, and associated comorbidities. Fluid replacement was done based on Parkland formula (4 mL/kg/per percent of burns), using crystalloid (Ringers lactate). Half of the fluid calculated was given in the first 8 hours postburn and the balance over 16 hours. The rate of fluid administration was aimed at maintaining hourly urinary output of 30 to 50 mL.

Following adequate resuscitation, we administered anti-tetanus drugs routinely except in children with reliable immunization history. Pain control was achieved by intravenous analgesia (pentazocin). Systemic antibiotics were not used routinely except when there was inhalational injury, evidence of wound infection, and in those admitted with soiled wound from domestic first aid or other infections. Wounds with evidence of infection were swabbed for culture and sensitivity and systemic antibiotics administered based on sensitivity reports.

The initial care of burns wound was by debridement done under general anesthesia or intravenous analgesia. Irrigation was carried out with copious amount of isotonic sodium chloride and cetrimide (1%), used for irrigation in some patients whose wounds were soiled from materials used as first aid. Wounds of the face and perineum were treated by exposure method using topical honcrivine (honey and acriflavine). Wounds involving the other areas were dressed, subsequent dressings were done after Hubbard's (saline) bath. Patients with circumferential full-thickness burns had escharaotomies. Full-thickness burns were excised with skin grafting.

Enteral feeding was commenced after 24 hours, except when there was gastric dilatation and or paralytic ileus. Later, a nasogastric intubation was done and H_2_ antagonist (cimetidine or ranitidine) administered. Physiotherapy was instituted early, including appropriate splints to prevent contractures. We consulted the ophthalmologist for injuries that involved the eye.

## RESULTS

### Incidence

Fifty-nine patients (18 in 2005, 21 in 2006, and 20 in 2007) were admitted and managed for burns. During this period, 4391 emergencies were seen at the accident and emergency unit, out of these 1654 (37.7%) were due to trauma. The 59 patients with burn injuries accounted for 3.6% of trauma injuries.

## AGE/SEX DISTRIBUTION

Table 1 shows the age/gender distribution. There were 33 males and 26 females (M:F = 1.3:1) whose ages ranged from 15 to 70 years (mean 29.4 years). The peak age group was in the 3rd decade. There were 56 children admitted for burns during this study period.

## TYPE/CIRCUMSTANCE/TIME OF BURNS

The etiology and gender distribution of burns is as shown in Table 2. There were 48 (81.3%) patients that sustained flame burns, out of these 41 patients were related to petroleum products (kerosene and petrol). Chemical burns that involved 7 (11.9%) patients, ranked 2nd (including a water board staff who sustained burn injury from soda lime). The motives behind the chemical injuries were robbery that involved 2 males and land dispute that involved another 2. Conflict over love affair was the motive behind the chemical burn in 2 female patients. Two patients (3.4%) sustained burns from molten iron while at work at a factory located at the Export Processing Zone, in Calabar.

The flame injuries from kerosene lantern/stove explosion in 19 patients were commoner in females (14 females and 5 males), and were frequent during the onset of darkness (between 6.00 PM and 8.00 PM). The flame injuries from petrol recorded in 22 patients were commoner among the males (17 males and 5 females). Eight males and 2 females sustained injuries as a result of road traffic accidents. The other injuries (7 males and 3 females) were domestic, from illegal storage of petrol at home. Two male cashiers suffered flame injuries at work during explosions at petrol stations.

## DOMESTIC FIRST AID

Thirty-seven (62.7%) patients were offered first aid, whereas 22 (37.3%) had no form of first aid before arrival. There were 7 patients who used cold water, 11 patients used raw eggs, 9 patients used palm oil, 5 patients used engine oil, and local herbal preparations were used in 5 patients.

## TREATMENT

Five patients with deep dermal burns (2 chemical and 3 flame burns) were offered skin grafting. Others had conservative treatment (debridement and dressing with honey). The latter included some patients with deep dermal burns, who could not afford to pay for surgery.

## OUTCOME

Satisfactory healing (absence of contracture and hypertrophic scar) was recorded in 28 (47.5%) patients. They were seen monthly for 6 months, on an average, in the outpatient department to monitor the progress of healing. Seven (11.9%) left against medical advice on account of poor finances. Table 3 shows the morbidity and mortality profile. Thirteen (22%) patients developed wound infection following burn, contractures were seen in 6 (10.2%) patients, and hypertrophic scar in 2 patients (3.4%). Fatal outcomes were recorded in 15 (25.4%) patients. Table 4 shows the relationship between the total burns surface area (TBSA%) with fatal outcomes and comorbidities. The outcomes of all the patients with TBSA greater than 60% were fatal and included a pregnant (24 weeks) woman. Out of the fatal outcomes in patients with TBSA, 21% to 40%, 2 patients had inhalational injuries, others with comorbidities included 1 with cerebrovascular accident and an epileptic who was also a diabetic.

## DISCUSSION

Burn injuries include coagulative necrosis of the skin. It may be caused by heat, cold, chemical, and electrical injuries.^6^ It affects approximately 1% of the population each year.^4^ They are common presentations to many health institutions in Nigeria^3^ and accounted for 3.6% of traumatic injuries seen in this center during the study period. The incidence varies from center to center. In Ibadan (Nigeria), 100 cases per year were reported,^6^ in Cairo (Egypt), 150^7^ and in Malawi, 304 yearly.^8^ The annual incidence in our center was 38 per year (59 adults and 56 children in 3 years). This low figure may have been due to a lower adherent population when compared with Ibadan; furthermore, some burn patients were seen at other health facilities within Calabar.

The age incidence also varies, adults were 59 (51%) with 56 (49%) children, with a ratio of 51:49. Other reports were, Ibadan (Nigeria) 62:38,^6^ Cairo (Egypt) 61:39,^7^ whereas Malawi, reported the involvement of more children and ratio of adults to children was 33:67.^8^ The peak age incidence was the 3rd decade (mean age 29.4 years). This is not surprising because this is the age when people are active, aggressive, and prone to trauma.

The epidemiology of burns varies from one part of the world to another and even in the same environment over time. It is a function of civilization, industrialization, culture, and societal stability.^3^,^5^ Flame burns were the commonest type of injury (81.3%), mostly from petrol and kerosene, whereas chemical burns ranked 2nd (11.9%). There were 2 industrial accidents that resulted in burn injuries from molten iron, this is as a consequence of industrialization and highlights the need for safety apparels and precautionary measures at work. Linares and Linares^9^ stated that for an approach for burn prevention most likely to be effective in a particular area, it should be based on knowledge of the prevalent etiological pattern of burns injury. The female preponderance to kerosene flame injury is due to the domestic inclination of the female gender. Kerosene lantern/stove should only be lit after fuelling as it is not uncommon to have adulterated kerosene with a high flash point. The male preponderance of flame injury from petrol is also not surprising. As a result of the high unemployment rate among the youths coupled with the scarcity of petroleum products, most males make brisk business in the illegal sale of petrol often conveyed in vehicles alongside passengers. Petrol is also stored at home.

Topical application of raw eggs, palm, and engine oil is a problem faced in our setting. It increases the cost and duration of debridement as well as interferes with the evaluation of the depth of burn. It may also increase the risk of infection. Public enlightenment on the use of cold water and early presentation is advocated.

Most of our patients, including some who had deep dermal burns, were offered conservative wound treatment as they could not afford to pay for excision and skin grafting. To reduce surface contaminants we use topical honcrivine. Honey plays 3 major roles in treating burn wounds and these include antimicrobial role because it contains inhibine, desloughing, enhancing granulation tissue formation, and epithelisation.^6^,^10^ It is also easily available and cheap. Inability to fund their treatment that is often expensive, was a problem faced by many patients and accounted for some of the complications.

All the patients with TBSA greater than 60% had fatal outcomes; this underscores the need for prevention. Poverty, unaffordable treatment options, and occasionally unavailable antimicrobials and dressings were problems faced by patients. The establishment of burns support groups dedicated to publicity on prevention as well as provision of financial aid for the treatment of victims is recommended.

Avoidance of refilling lanterns/stoves when lit, careless handlings of petroleum products, and illegal possession of dangerous chemicals were notable risk factors. Education of the public on burns prevention using the electronic and print media, schools, religious organizations and health workers on the basis of areas of risk highlighted in this study are recommended.

In conclusion, the establishment of burn support groups, health education, improved funding, and provision of modern medical care facilities for burns would improve the overall outcome.

## Figures and Tables

**Table 1:** Age and gender distribution*

	Gender		
Age range, y	M	F	Total (%)
1–10	. . .	. . .	. . .
11–20	4	7	11 (18.6)
21–30	20	8	28 (47.5)
31–40	5	6	11 (18.6)
41–50	4	2	6 (10.2)
51–60	. . .	2	2 (3.4)
61–70	. . .	1	1 (1.7)
	33	26	59 (100)

* Age range 15–70 years (mean 29.4 years).

**Table 2:** Etiology and gender distribution

	Gender			
Etiology	M	F		Total (%)
**Fluid**	. . .	2		2 (3.4)
Hot water				
Molten	2	. . .		2 (3.4)
**Flame**				
Kerosene	5	14	19	
Petrol	17	5	22	48 (81.3)
Gas	3	3	6	
Candle	1	. . .	1	
**Chemical**				
Acid	4	2	6	
Alkali	1	. . .	1	7 (11.9)
**Total**	33	26		59 (100)

**Table 3:** Morbidity and mortality

	No.	Total (%)
**Wound infection**		
Flame	8	13 (22)
Chemical	5	
**Tetanus**		
Flame	1	1 (1.7)
**Contractures**		
Flame	2	6 (10.2)
Chemical	4	
**Hypertrophic scar**		
Flame	2	2 (3.4)
**Fatal outcome**		
Fluid (epileptic)	1	15 (25.4)
Flame	14	

**Table 4:** Total burns surface area (TBSA) and mortality

TBSA, %	%	Fatal outcomes	Remarks
1–20	27	. . .	. . .
21–40	19	5	Inhalational injury, 2; cerebrovascular accident, 1; epileptic or diabetic, 1
41–60	5	2	Epileptic 1
61–90, 1 pregnant	6	6	
91–100	2	2	–
